# Home-Care Nurses’ Experiences of Caring for Older Adults With Type 2 Diabetes Mellitus and Urinary Incontinence: An Interpretive Description Study

**DOI:** 10.1177/23779608211020977

**Published:** 2021-06-08

**Authors:** Melissa Northwood, Jenny Ploeg, Maureen Markle-Reid, Diana Sherifali

**Affiliations:** 1 School of Nursing, McMaster University, Hamilton, Ontario, Canada; 2 Diabetes Care and Research Program, Hamilton Health Sciences, Ontario, Canada

**Keywords:** nursing assessment, home care services, aged, multiple chronic conditions, diabetes mellitus, urinary incontinence, social determinants of health, quality of life, complexity, interpretive description

## Abstract

**Introduction:**

A third of older adults with diabetes receiving home-care services have daily urinary incontinence. Despite this high prevalence of urinary incontinence, the condition is typically not recognized as a complication and thereby not detected or treated. Diabetes and urinary incontinence in older adults are associated with poorer functional status and lower quality of life. Home-care nurses have the potential to play an important role in supporting older adults in the management of these conditions. However, very little is known about home-care nurses’ care of this population.

**Objective:**

The objective of this study was to explore how nurses care for older home-care clients with diabetes and incontinence.

**Methods:**

This was an interpretive description study informed by a model of clinical complexity, and part of a convergent, mixed methods research study. Fifteen nurse participants were recruited from home-care programs in southern Ontario, Canada to participate in qualitative interviews. An interpretive description analytical process was used that involved constant comparative analysis and attention to commonalities and variance.

**Results:**

The experiences of home-care nurses caring for this population is described in three themes and associated subthemes: (a) conducting a comprehensive nursing assessment with client and caregiver, (b) providing holistic treatment for multiple chronic conditions, and (c) collaborating with the interprofessional team. The provision of this care was hampered by a task-focused home-care system, limited opportunities to collaborate and communicate with other health-care providers, and the lack of health-care system integration between home care, primary care, and acute care.

**Conclusion:**

The results suggest that nursing interventions for older adults with diabetes and incontinence should not only consider disease management of the individual conditions but pay attention to the broader social determinants of health in the context of multiple chronic conditions. Efforts to enhance health-care system integration would facilitate the provision of person-centred home care.

Urinary incontinence (UI) is a prevalent problem internationally for older adults living with type 2 diabetes mellitus (T2DM) who receive home-care services (Hsu et al., 2014; Vetrano et al., 2016). UI in older adults (aged ≥65 years) is associated with many negative outcomes, such as falls, fractures, anxiety, depression, reduced quality of life, and premature institutionalization (Hsu et al., 2014; Coyne et al., 2013; Wagg et al., 2017). A cross-sectional study of older adults with T2DM receiving home-care services in Ontario, Canada, found that 33.7% experienced daily UI (Northwood et al., 2021a). Despite this high prevalence of urinary incontinence in older adults with diabetes, the condition is often not recognized as a complication and thereby not detected or treated (American Geriatrics Society, 2013; Brown et al., 2005). The majority (90%) of older adults with T2DM receiving home-care services are also living with multiple (≥2) chronic conditions, which contributes to poorer diabetes self-management and increased health-care utilization (Gruneir et al., 2016).

Home-care programs internationally are caring for older clients with increasingly complex health- and social-care needs (Canadian Institute for Health Information, 2018; Martinsen et al., 2018). Home care in Canada encompasses acute, rehabilitation, long-term support, and end-of-life services provided in the home and community setting (Better Home Care in Canada Partners, 2016). Home care is not mandated as part of the universal health care system of Medicare and provincial governments have organized home care provision of nursing, therapies, and personal support through a combination of public, voluntary, and commercial providers (Baranek et al., 2004). In the province of Ontario, at the time of the study, home-care services were managed by 14 local health integration networks and contracted out to multiple service providers (Donner et al., 2015). Home-care coordinators, most typically nurses, employed by these networks assess client eligibility for services and monitor and adjust their service plans over time. These coordinators would make referrals to service provider for nursing care, including for management of incontinence.

As such, home-care nurses play an important role in supporting older adults in managing their UI (Community Health Nurses of Canada, 2019). However, no research was located on how home-care nurses care for older adults with T2DM and UI. Three studies were found that described the experiences of home-care nurses caring for older adults living with diabetes (Fox et al., 2006; Gifford et al., 2013; Kolltveit et al., 2018). Home-care nurses’ in these studies experienced many barriers to caring for older adults with diabetes, such as limited access to other home-care providers (i.e., registered dietitians or specialty diabetes educators), lack of formal collaboration with primary care, and clients’ social, mental, and physical concerns that needed management in addition to providing diabetes care (Fox et al., 2006; Gifford et al., 2013; Kolltveit et al., 2018). Only one study was found regarding home-care nurses’ experiences caring for older adults with UI (Jansen et al., 2013). In this grounded theory study of home-care providers’ knowledge translation related to UI, nurse participants focused on developing a relationship with the client and their caregiver but did not describe sharing continence-promoting knowledge (Jansen et al., 2013).

Thus, little is known about how nurses care for older adults with T2DM and UI. Understanding the subjective experiences of nurses is an important part of intervention development to clarify the clinical problem and identify effective strategies to mitigate the problem as well as gaps in service delivery (Sidani & Braden, 2011). This study will address this gap in knowledge and create a much-needed evidence base to inform home-care nursing practice, policy, and future research. The research question for this study was: How do home-care nurses care for older adults with T2DM and UI?

## Methods

### Design

This research question was addressed using an interpretive description methodology. The first author and study leader was a PhD student and home-care nurse at the time of the study. The impetus for exploring this topic was related to a lack of research evidence to inform nursing interventions for this population and the very challenging clinical dilemmas experienced by the first author in supporting her older home-care clients with T2DM to achieve continence. These challenges included the lack of health-care providers’ knowledge of the relationship between T2DM and UI in older adults and the subsequent under-treatment of UI.

This study was part of a convergent, mixed methods research study with the aim of better understanding the complexity of living with T2DM and UI in older adults receiving home-care services (protocol previously published; Northwood et al., 2019; Creswell & Plano Clark, 2018). The mixed methods study also involved a qualitative exploration of the experiences of older adults with T2DM and UI receiving home-care services (Northwood et al., 2021b) and a quantitative strand to determine the prevalence and correlates of UI in this population (Northwood et al., 2021a). Interpretive description is an applied health research approach that identifies a knowledge-practice gap and gathers knowledge to achieve a contextual understanding of the commonalities and differences of persons experiencing the phenomena in question (Thorne, 2016). This paper presents analysis not covered in the other publications.

A model of complexity for persons with multiple chronic conditions was used to inform study design, develop the interview guide, and inform the initial phase of analysis (Grembowski et al., 2014; Thorne, 2016). This model of complexity was chosen as it considers many factors that lie outside of the health-care system affecting older adults’ health and well-being, such as community services and social support (Zullig et al., 2015). Clinical complexity is influenced by the capacity of the health-care system to meet clients’ needs based on their individual characteristics, health and well-being, and social supports as well as contextual health, economic, and social policies that create health disparities (Grembowski et al., 2014).

### Setting and Sampling

Nurse participants were purposively sampled from three local health integration networks in southern Ontario (Hamilton Niagara Haldimand Brant, Mississauga Halton, and Waterloo Wellington). These networks were chosen given their proximity to the research team to facilitate in-person interviews. Nurses, by virtue of their clinical experiences and observations of many older adults with both T2DM and UI, provide the “thoughtful clinician” perspective and can provide rich insights into this population and the care they require given their encounters with many clients (Thorne, 2016, p. 93). For this reason, the target sample size was 10 to 15 nurses (Malterud et al., 2016). Maximum variation sampling was used to ensure diversity in role (e.g., continence specialist), professional registration class (e.g., registered nurse), and length of experience in home care among the study participants (Patton, 2002). Participants met the following inclusion criteria: licensed as registered nurses (general or extended class) or registered practical nurses; practiced in home care for at least six months; and cared for older adults with T2DM and UI. Recruitment occurred from July 2017 to May 2018. Nurses were recruited through one service provider’s electronic newsletter and one of the authors emailed nurses in her professional network informing them of the study.

### Ethical Considerations

This study was approved by the Hamilton Integrated Ethics Review Board (project #3024-C) and followed the guidelines of the Tri-Council Policy on the ethical conduct of research (Tri-Council, 2014). Informed, written consent or verbal consent (for phone interviews) was obtained from all participants prior to conducting the interviews. Participants’ privacy and confidentiality was guarded by password protecting and storing all digitally recorded interviews and transcripts on a password protected server, and anonymizing the transcripts of identifying information (e.g., names and locations).

### Data Collection

Interviews were conducted over the phone or in-person at a location of the participants’ choosing (e.g., office) lasting between 45 and 90 minutes. The interviews were completed by the first author using a semi-structured interview guide beginning with the grand-tour question: “Can you tell me about how you approach the care of an older adult that has both diabetes and incontinence?” (Spradley, 1979). The interview guide was informed by research evidence and contained questions to address each of the components of the complexity model (refer to Table 1; Grembowski et al., 2014). Sociodemographic characteristics were also captured using a short demographic survey. The interviews were digitally audio-recorded and transcribed verbatim. Recruitment ended when the authors felt they could reasonably conclude that enough experiences were obtained in order to generate a credible interpretive description (Thorne, 2016).

**Table 1.** Semi-Structured Interview Guide.

Complexity model dimensions	Interview questions
Person/health	Tell me about some experiences you have had caring for an older adult that has both diabetes and incontinence. Can you tell me about how you approach the care of an older adult that has both diabetes and incontinence? Would you describe for me how you decide what to do for a client with both diabetes and incontinence? Can you describe for me what the functional abilities are like for your typical client with both diabetes and incontinence? From your experience, what nursing interventions work best for an older adult with both diabetes and incontinence? Thinking about the clients you see in your practice, do you think their income [and gender] affects how they managed their diabetes and incontinence? If you were supervising a nursing student in the home environment, what would be the most important lessons you would want to tell her (or him) about working with older adults with diabetes and incontinence?
Social support	Tell me about what it is like working with older adults’ caregivers, such as the spouse or child? How does this social support influence how your older adult clients manage their diabetes and incontinence?
Community resources	Thinking about the clients you care for with diabetes and incontinence, tell me about what it is like working with other home care workers, such as their PSWs or care coordinators. Can you give me an example? Could you tell me about how you approached your client’s care in this situation? If you were in charge of home care and could decide how to organize care, tell me about any changes you would make in how older adults with diabetes and incontinence are cared for in their homes?
Health system	Thinking about older adults with incontinence and diabetes, tell me about a time when you worked with your client’s primary care physician [or nurse practitioner]? Could you tell me about a time when one of your clients with diabetes and incontinence had to go to the hospital? What happened? What did you do? Can you tell me all the different steps you took to see them from the home to the hospital?
Conclusion	Is there anything else that we haven’t talked about that you think would be important for me to know about your experiences providing home care to older adults with diabetes and incontinence?

### Data Analysis

Data analysis occurred concurrently with data collection using an inductive and iterative process (refer to Table 2). The interpretive description analytical process involved constant comparative analysis, reflexive journaling, attention to commonalities and variance, and research team discussion (Thorne, 2016). The first two phases of the analysis process were conducted by the first and second authors, utilizing both handwritten coding and a word-processing program to describe and rework codes into themes. During the early “sorting and organizing” phase, the components of the complexity model were used to organize the data (Grembowski et al., 2014; Thorne, 2016, p. 156). The thematic outputs were discussed with all authors at four time points and culminated in the third stage of “transforming the pattern in findings” (Thorne, 2016, p. 173). The audit trail was shared at meetings, showing the development of sorting the data, the coding scheme and associated quotes, interpretative themes, and reflections from journaling (Thorne, 2016). Further analysis and refinement of the written conceptualization continued with input from all authors to produce these final written results.

**Table 2.** Interpretive Description Process of Analysis.

(1) “Sorting and organizing” by reading and re-reading transcripts and field notes, listening to digital recordings, sorting data by components of the complexity model, journaling preliminary thoughts.
(2) “Making sense of pattern” by applying descriptive codes to segments of the transcripts and pattern codes to group together descriptive codes into larger themes
(3) “Transforming pattern in findings” by testing relationships of themes to emerging conclusions and ensuring no other explanation
(4) Writing conceptualization of the findings

*Note*. Adapted from Thorne (2016).

### Trustworthiness

To ensure trustworthiness, credibility processes were built into the study design (Thorne, 2016). First, the research team had expertise in qualitative research, mixed methods research, and nursing intervention research with older adults, caregivers, and persons with diabetes. While the first author conducted all of the interviews, the entire team reviewed a set of transcripts. The second author also participated in coding a number of transcripts and regular meetings with the first author to reflect on the emerging findings. Developing themes and supportive quotes were shared with team over the analytical process to reach consensus on the main themes. Aligned with the principles of interpretive description, the analytical process to arrive at the main themes is our best efforts at constructing a “probable truth” that holds disciplinary relevance (Thorne, 2016, p. 238). Second, given that the first author was a home-care nurse, the research team was intentional about challenging if her impressions were reflective of her experiences or those of the participants. Decisions were documented in meeting minutes, serving as an audit trail. The first author also kept a journal to reflect on interviews, team discussions, and developing findings. Third, sampling included diverse participants and in the analytical process, we attended to both commonalities and differences in experiences. Finally, these qualitative results were reported in adherence to COREQ criteria (Tong et al., 2007).

## Findings

### Description of the Nurse Participants

Fifteen nurses participated in the interviews (nine registered nurses, one nurse practitioner, and five registered practical nurses; see Table 3). Almost all (93%) of the participants were female and were an average of 54 years of age. Almost two thirds (66.7%) of study participants had more than 16 years of clinical experience and over one half (50%) had more than 11 years of experience working in home care specifically. Forty percent of participants had completed formal education as a nurse continence advisor, and 13.3% had completed specialized education in wound, ostomy, and continence care. They were employed by 5 different home-care providers.

**Table 3.** Nurse Participants’ Demographic Characteristics (N = 15).

Variable	n (%)
Gender	
Male	1 (6.7)
Female	14 (93.3)
Education	
Diploma	9 (60.0)
Bachelor’s degree	5 (33.3)
Master’s degree	1 (6.7)
Registration	
Registered nurse	9 (60.0)
Registered practical nurse	5 (33.3)
Nurse practitioner	1 (6.7)
Certificate	
** ** Nurse continence advisor	6 (40.0)
** ** Nurse specialized in wound, ostomy and continence	2 (13.3)
** ** None	7 (46.7)
Years in practice	
** ** 6–10	3 (20.0)
** ** 11–15	2 (13.3)
** ** 16–20	1 (6.7)
** ** 20+	9 (60.0)
Years in home care	
** ** 1–5	3 (20.0)
** ** 6–10	4 (26.7)
** ** 11–15	2 (13.3)
** ** 16–20	4 (26.7)
** ** 20+	2 (13.3)
	Mean (standard deviation)
Age	53.8 (13.7)

### Home-Care Nurses’ Care of Older Adults With Diabetes and Urinary Incontinence

**Figure 1.**
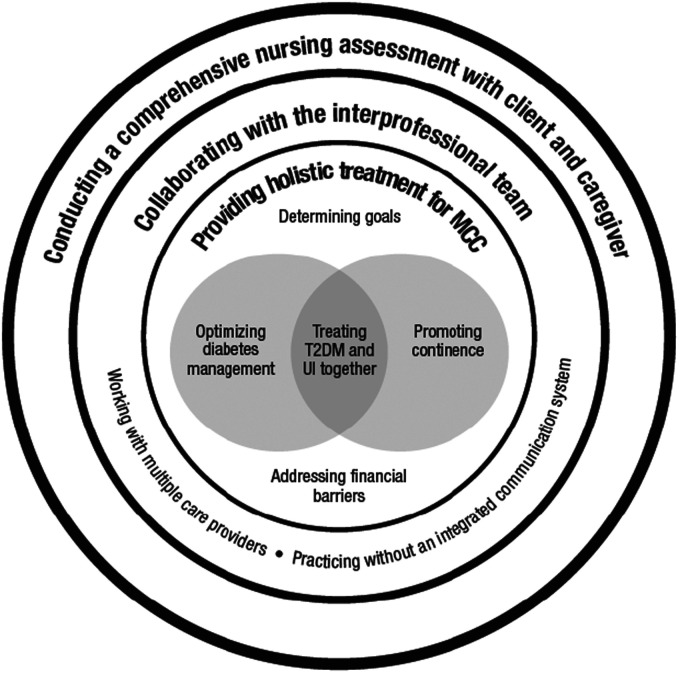
Thematic Conceptualization of the Findings.

Three main themes and associated subthemes emerged regarding how home-care nurses cared for older adults with T2DM and UI: (a) conducting a comprehensive nursing assessment with client and caregiver, (b) providing holistic treatment for multiple chronic conditions, and (c) collaborating with the interprofessional team (see Figure 1).

#### Conducting a Comprehensive Nursing Assessment With Client and Caregiver

Participants described that the first step in caring for older clients with T2DM and UI and their caregivers was to conduct a comprehensive nursing assessment. They revealed that the contributing factors to T2DM and UI management were quite complex owing to the presence of multiple chronic conditions. As this participant detailed:

A lot of people with diabetes also are obese, high blood pressure, their blood sugars are kind of not in control, peripheral edema, and neuropathy affecting their bladder and deceased mobility. Like there’s just so many contributing factors than besides being, let’s say, an older woman with five kids. People with diabetes have so many other contributing factors that they are very complex patients. (03)

As a result, nurse participants not only collected a history related to T2DM and UI but also on their other chronic conditions, geriatric syndromes (e.g., falls), and self-management abilities: “You have to do a comprehensive assessment first before you make any recommendations including their functional abilities, cognitive deficit, mental health issues, social supports, of course, the impact of incontinence on quality of life to determine your approach” (02). Nurses also reported collecting information on medications, falls, involvement of specialists, loneliness, diet, and fecal incontinence. The participants shared that many of their clients had some degree of cognitive impairment that may or may not be formally diagnosed and this was important for nurses to ascertain as their care would have to be tailored to accommodate memory impairment. This nurse explained:

When there is cognitive impairment involved, then we need to work more with the people that are around that patient and we need to assess as to what degree does this patient receive support in order to be able to function in the community independently or semi-independently. (01)

Nurses evaluated the home environment for condition of the home (i.e., disrepair, safety), food security, and distance to the bathroom. Being in the home afforded the nurses key insights, as this participant described:

Because in community nursing, you’re in there, you see that there’s only a chunk of cheese in the fridge or that they can’t get from their bathroom to their chair without leaking because they’re going up three flights of stairs. (09)

Nurse participants indicated that they also assessed the needs of caregivers of the older adults with diabetes and UI. This helped them to determine what support they were providing to older adults, such as maintaining hygiene or seeking health care, and the kind of support that they needed. As well, nurse participants identified the impact of caregiver burden (e.g., sleeplessness, frustration) and planned strategies to lessen their caregiving duties (e.g., respite services). The nurse participants regarded supporting caregivers in managing UI to be of the upmost importance in facilitating aging-in-place, as described by this nurse: “And a lot of them will talk about placement, because they can’t handle the urinary incontinence or bowel incontinence” (09).

Participants also assessed areas specific to T2DM self-management (e.g., blood glucose monitoring, using medications as directed, making healthy food choices). Nurse continence advisor participants determined the relationship between hyperglycemia and UI and the client’s perceptions regarding this relationship, as well as standard UI assessment components (e.g., fluid intake, voiding frequency, physical assessment). A notable exception was raised by nurses specializing in wound care, who shared that incontinence was not part of their standardized home-care assessment: “I leave it to the patient to bring it to my attention” (NP10).

#### Providing Holistic Treatment for Multiple Chronic Conditions

Nurse participants identified several strategies that they used to manage T2DM and UI. Some of these strategies improved both T2DM and UI simultaneously, some addressed broader concerns (i.e., financial barriers), and others targeted each condition separately. The nurses indicated that they worked diligently to try to provide holistic care however, they faced several barriers.

#### Determining Goals

The nurse participants took a pragmatic approach to determining goals for treatment with older adults and their caregivers. They described establishing what bothered the client most and what could realistically be improved given the presence of multiple chronic conditions and planned accordingly. This nurse participant described goal setting and how it was used to engage patients in self-management activities:

Often their main concern is the embarrassment of incontinence. They will cut themselves off from going out or spending time with family and friends and doing anything physical because they are so nervous about the incontinence issue. And so just saying that with this change in lifestyle and some of the small changes [drinking more water], they can really get a better quality of life and spend more time with grandchildren enjoying them, and that gets it really high on their interest list. (02)

The goals were based on what the client could and wanted to do. Nurses also elicited caregiver input on feasibility and practicality of goals. For some of the participants’ clients, continence was not the goal but rather a reduction in product use, incontinence episodes, or nighttime voids: “If people have many complex comorbidities then you may decide to help them manage the incontinence and not actually make any suggestions for improvement” (03).

#### Treating T2DM and UI Together

The participants explained that they provided education to the older adult, family caregiver, and personal support worker if involved on UI, diabetes control, and the relationship to other chronic conditions. They often described how they had to address the older adults’ lack of recognition of the relationship between T2DM and UI, as this participant described: “One would have to really do quite a bit of teaching to get them to connect the dots” (02).

The participants described several other strategies, such as supporting older adults to engage in physical and social activities as a distraction from health problems related to multiple chronic conditions, and changes to dietary and fluid intake (i.e., improving clear fluid intake, increasing dietary fibre). Participants felt that interventions to attain optimal, personalized glycemic control would improve both T2DM and incontinence.

#### Addressing Financial Barriers

Participants indicated that many older adults faced financial barriers to adequate care of T2DM and UI: “You get a senior on like CPP [Canada Pension Plan], they can’t even afford incontinence pads, let alone better foods” (05). Due to the high cost of incontinence products, the participants witnessed many clients using homemade incontinence products that put them at risk for skin breakdown and infection. As such, nurse participants would try to intervene by connecting older adults to programs that offset the costs of incontinence products.

Living well with diabetes was described as financially impossible for some clients served by the participants:

Are they actually able to afford and purchase the food items that we are suggesting they take? Are they actually able to get these items that are high in fiber, high in protein, low in sugar and all that? So not just nutritional things but also if I’m advocating and recommending that they get their toenails trimmed and looked after by a foot care nurse. How does that impact the oftentimes fixed income that they have because it’s not the only expenditure that I’m suggesting to them? (13)

Managing T2DM with insulin was also very costly for older adults. A few nurses educated clients on how to access provincial funding for diabetes supplies (e.g., needles and testing strips). Participants provided other examples of financial barriers to clients’ overall self- management, including transportation to medical appointments and purchasing home-care services or retirement-home living. However, not all nurse participants felt it was their role to address their clients’ financial barriers.

#### Optimizing Diabetes Management

Nurse participants used multiple interventions to manage T2DM. They believed that many older adults may not have had or did not recall previous diabetes education: “I think with the older adult, because they probably have had it for years and years, probably years ago when they were first diagnosed, the emphasis on education on diet probably wasn’t as strong as it today” (05). Nurses also raised concern that many older adults do not monitor their blood glucose even when that information would inform their self-management: “They’ve been to the diabetic clinics before in the beginning and they’ve been shown what to do, but complacency sets in and a lot of times they just have so many other comorbidities that diabetes is kind of low on the scale” (09). Furthermore, the participants observed that some older adults did not perceive exercise as a component of self-management for T2DM.

Thus, participants provided education to clients and their caregivers on monitoring their diabetes and preventing diabetes-related complications: “I saw a gentleman today, he’s got severe neuropathy, and he didn’t know that he should be checking his feet every day. That’s a pretty basic thing to know” (15). The nurse participants described making referrals for older adults to outpatient or in-home (much less available) diabetes education programs: “If people are able to get out and interested in their disease, there are enough classes that they can actually go to and learn more about it” (14).

The nurse participants also connected with primary-care physicians to address suboptimal diabetes control. This was most challenging when clients were homebound related to other chronic conditions, as this nurse explicated:

How can we advocate for these things to happen because in home care there’s a lot of obstacles to getting them done? Say for example, the lab tests, how can we get them every three months, if they’re not really mobile and they’re stuck at home, how can we advocate with the help with the LHIN [local health integration network] to have the home-care lab done so that the doctor actually has the results to be able to determine where to go from there? (13)

Nurse participants identified that some health-care providers under-treated hyperglycemia in older adults due to the belief that tighter control was not required or due to concern for risk of hypoglycemia in frail older adults with multiple chronic conditions.

#### Promoting Continence

Clients who were living with UI for a long time also presented unique challenges to the nurse participants. Many older adults had not received proactive or preventative treatment for UI at the onset. Nurses indicated that older adults often felt that UI was not treatable and an unfortunate consequence of aging, a misconception reinforced by health-care professionals. These long-held beliefs were also complicated by the stigmatizing nature of UI, as elucidated by this participant:

Urinary and fecal incontinence, for some people, is a very embarrassing scenario, either that or it’s a scenario where they assume that being incontinent, whether it’s the bladder or bowel, is part of the aging process. Have been told by their doctor, “Well, you’re getting older and it’s just part of your life and you’re just going to have to live with it,” which still happens as of today. (01).

This nurse continence advisor participant argued that home care should have greater emphasis on treating UI: “I would certainly intervene with continence more because it affects people’s quality of life and their interaction with family and friends and so that is a huge implication that they [home-care decision makers] don’t recognize.” (09). Interventions to manage UI described by the nurse continence advisor participants were comprehensive and included: recommending products, educating on urinary tract infection prevention, assessing and treating fecal incontinence, resolving constipation, teaching pelvic floor muscle exercises, recommending adaptive equipment (e.g., commode, urinal), reduction of caffeine intake, and advocating to primary-care physicians on de-prescribing medications contributing to UI, such as sedatives. For clients with dementia, the participants described troubleshooting with personal support workers and caregivers regarding challenging behaviours when changing incontinence products and toileting.

Participants identified that they would request a nurse continence advisor referral through the home-care coordinator or reach out directly to a nurse continence advisor for advice if one was employed by their agency. Some of these participants shared that they did not have knowledge of continence promotion strategies to be able to independently intervene:

So oftentimes because my role was focused on diabetes—although it does go into a little bit of foot care and incontinence as well—I find that I don’t often give out advice [about UI]. Just because, first of all, my knowledge, I don’t think, is to the point where I could be giving out advice before I consult someone else. (13)

Nurses indicated that their ability to provide holistic care for T2DM and UI was often challenging due to the current focus on short-term, post-acute care within the home-care sector. As this participant revealed:

So, it used to be that they [home care] looked after seniors primarily in the home and so people went into hospital, got discharged, and came home. And now the LHIN [local health integration network] is being mandated to get people out of the hospitals faster. So, the hospitals are wanting to shuffle people out and I think that those pressures are huge and I think that that’s why they’ve [home care] gone to this sort of task-orientated care. (09)

This task orientation was due to changes in “reason for referral” from home-care coordinators—such as wound care or catheter care—and the allocation of a limited number of visits. This nurse explained that she no longer had clients on long-term service for chronic disease management:

It is a big change and I don’t think that was for the better. We would have the people that we had on for monitoring that we saw once a week [but now] you’d get them back on [service] because they’d been in the hospital because nobody made that phone call [to the physician] to say “Hey, Mrs. Jones’s feet are getting puffy, could we increase her Lasix?” (12)

Participants noted that short visit lengths and being paid by the visit (rather than by the hour) contributed to task-focused, rather than holistic, person- and family-centred care. Many of the participants were caring for older adults with diabetes who also had ulcers. They shared feeling rushed in a 30-minute visit that included travel, documentation, care, and education: “It provides a challenge but at the same time you can’t ignore the client’s needs” (11). These time pressures had negative consequences, such as keeping conversation to a minimum and focusing on the task rather than other pressing client concerns:

I think it’s the system that perpetuates the “get it done fast” and the focus is on the task instead of on the person. So, let’s pay this half an hour to do a dressing and you can still address diet and that kind of stuff while you are doing that dressing but people are in and out so fast that the client doesn’t have time to tell them that their daughter died last week. (09).

#### Collaborating With the Interprofessional Team

Given clients’ multiple chronic conditions, they often received care from multiple providers (e.g., primary-care physician, physiotherapist, occupational therapist) across multiple settings (e.g., home care, acute care, primary care). While nurse participants identified the value of collaborating with the interprofessional team, they indicated that they had few opportunities to do so.

#### Working With Multiple Care Providers

When nurses worked with other interprofessional team members, they found this incredibly valuable to supporting care of this population. For example, some nurses shared that, over time, they had developed trusting relationships with primary-care physicians and these physicians valued and relied on their expertise and updates on clients. As well, one nurse continence advisor participant highlighted the value of collaborating with personal support worker: “I really like working in collaboration with the PSWs. They’re my eyes when I can’t be there” (08). Some nurse participants felt that their clients benefited when they collaborated with occupational therapists regarding home safety assessments to prevent falls and with dietitians about nutritional strategies to live well with diabetes.

However, participants felt that they had limited opportunities to collaborate with other home-care providers. They described a “disconnect” between service providers as they worked with different agencies and out of different offices (13): “Most of the time it’s through the patient’s word: ‘the OT [occupational therapist] told me this’, ‘the nurse told me that’” (10). The nurses shared that even when another home-care provider was involved, that professional’s willingness to be a team player determined the extent of the collaboration: “They [occupational therapists] don’t leave their reports in the home and a lot of the time I don’t even know who they are, I just know that they came” (11).

Also, the participants shared that there is no formal, built-in process to regularly collaborate with other providers both within and outside of home care. For example, this participant described her experiences attempting to collaborate with primary-care physicians:

Some of the physicians you call them, they call you right back, excellent discussion and things move ahead really well. Others, you call, and like I’m waiting for a physician to call me right now about somebody who actually has, I think, an infected diabetic ulcer. (14).

The participants reported that agency policies dictated that changes to a care plan be communicated to the personal support worker supervisor (registered nurse or registered practical nurse), who relayed the information to the personal support worker, not allowing for direct personal support worker -nurse collaboration.

#### Practicing Without an Integrated Communication System

The participants noted that access to and sharing of health information within home care and with other health-care settings was important in providing holistic, safe care and fostering team collaboration but was fragmented and uncoordinated. The participants reported that each home-care agency had its own health record for each client (paper or electronic). “The thing that would be ideal, if we had better ways to communicate with each other. You know, there’s no linkages still where we could communicate in the patient’s chart from the LHIN [local health integration network]. So, for example, if we could ever document so that we could all read the notes” (03). Participants also had inconsistent access to the home-care electronic health record. Participants explained how the home-care coordinator routinely completed a standardized, interRAI assessment with clients but this document was not accessible to all participants through the online, local health integration network-hosted platform. Some participants reported referring to the interRAI assessment in order to not duplicate assessments:

Ninety-nine percent of the time we get the RAI included with the referral and that just gives me an idea when I look at it, what I’m going to be walking into. Sometimes I’ll pull off data from it and put it onto my assessment [form]. (12).

However, most participants revealed that they did not understand the purpose of the interRAI assessment or how to interpret the information within this document: “I mean it was page, after page, after page of … If there was a way where they could take the yes/no answers and just spit them out on a page, but I never really saw a kind of summary of things” (14).

Participants did not have access to primary-care or acute-care electronic health records. Instead, the participants revealed that they expended time and energy daily on a cascade of work-arounds to access health information from primary care, acute care, and specialist physician providers. The participants revealed that they often had minimal knowledge of their clients’ health history and often relied on the client and caregiver accounts of what happened during a hospital admission or at specialist consultations. This participant summarized the barriers: “I think the electronic health record is the first step but it’s a long way to go yet. We need visibility to the doctor’s records, to the specialists, to the hospital, to the rehab facility” (12). This participant’s description of trying to obtain a urologist’s report elucidates the time-consuming process of obtaining relevant health information on her home-care clients:

The guy [client] said to me, “The urologist told me there’s nothing I can do.” Well, that doesn’t help me much. So, I phoned the family doctor and he goes, “We don’t have their records.” “Did you change doctors?” “Well, we were back and forth and we were in an apartment for a few months, we used that doctor.” I said, “Then you need to call that doctor and get his records from the urologist because the urologist won’t give it to me directly.” So, I’m in the process of tracking that paper to get it to the new family doctor so I can get a copy because I don’t know what to do with him. (05).

Participants felt that a system-wide shared electronic health record would improve communication and coordination of care for providers across all health-care settings.

## Discussion

To our knowledge, this is the first interpretive description study to explore how home-care nurses care for older adults with T2DM and UI. In summary, conducting a comprehensive nursing assessment with clients and their caregivers and providing holistic treatment for multiple chronic conditions was hampered by a task-focused home-care system, limited opportunities to collaborate with other health-care providers, and a lack of mechanisms to promote communication. The findings of this study generated several key contributions to an understanding of how home-care nurses care for this population.

To start, this qualitative study provides insight into the approach home-care nurses took in their assessment and care of older adults with complex needs. The findings contain detailed information about the synergistic strategies used to support older adults in managing their T2DM, UI, and multiple chronic conditions. How nurses cared for T2DM and UI together in older adults has not previously been described in nurse-led continence intervention trials (Albers-Heitner et al., 2011; Moore et al., 2003; Williams et al., 2005; Xu et al., 2018). In the home-care context, nurses recognized that UI results from the additive effect of impairments in multiple domains (Tinetti et al., 1995). This type of synergistic treatment of concordant conditions is recommended by multimorbidity researchers (Boyd & Fortin, 2010). The necessity to provide holistic treatment for T2DM and UI is supported by Hsu et al.’s (2014) study of the predictors of UI in older adult home-care recipients with T2DM. Geriatric factors, such as mobility and cognitive impairment, are as important to consider as diabetic factors in the management of UI (Hsu et al., 2014).

Second, a key finding of this study was in this home-care setting specialist nurses, who due to educational preparation and clinical experience, possess knowledge and expertise regarding continence promotion. Unlike research in other settings, where the majority of health-care providers did not know how to manage UI, participants in this study either had that expertise themselves or were aware of nurses with that expertise that they could consult with (i.e., requested a referral to a nurse continence advisor; French et al., 2017; Sui et al., 2001). These specialist roles exist internationally in home-care programs for example, in Australia, the United States, and the United Kingdom (Paterson et al., 2016). The generalist home-care nurses in this study self-identified learning needs regarding continence promotion strategies. This finding is consistent with a qualitative evidence synthesis that found education is an enabling factor for providers in implementing behavioural interventions for UI (French et al., 2017).

Third, the focus of home care on task-focused care was a barrier to the nurses’ ability to provide a holistic, person- and family-centred approach to care. Task- or disease-centric care has previously been noted as incompatible with person-centred care for older adults with multiple chronic conditions (Bernsten et al., 2019; Farmanova et al., 2019; Grembowski et al., 2014). Given the high prevalence internationally of older adults living with multiple chronic conditions requiring long-term support, there is need for a broader, health-care system-wide reorientation from a single task or “single condition paradigm” to a model of person- and family-centred care (Entwistle et al., 2018; Grembowski et al., 2014, p. S11).

Fourth, the nurses identified the need for improved collaboration among home-care providers and between home-care and primary-care providers in caring for older adults with T2DM and UI. Other research of Ontario home-care recipients noted primary-care physician home-care coordination billing codes were rarely used (only 3.9% of their clients), suggesting a lack of integration and collaboration between home- and primary-care providers (Jones et al., 2019). The benefits of interprofessional collaboration in the care of older adults with multiple chronic conditions is supported by research evidence and this absence of standard-operating procedures to facilitate collaboration is a significant care gap for this home-care population (Baxter & Markle-Reid, 2009; Boult et al., 2009). For example, a qualitative exploration of interprofessional home-care team members involved in a fall prevention intervention found that face-to-face, regularly scheduled communication through team meetings enhanced collaboration more than their typical asynchronous voice mail or email communication (Baxter & Markle-Reid, 2009).

Fifth, the finding of the need for formal mechanisms to improve communication and coordination of care across settings—home care, primary care, acute care—is noteworthy. A critical component of an integrated health-care system is an intersectoral electronic health record to improve communication and coordination of care (Bernsten et al., 2019; Blaum et al., 2018; Donner et al., 2015). Also revealed was that although the interRAI home-care standardized assessment has been mandated in Ontario since 2002, not all nurses had access to the interRAI assessments (Heckman et al., 2013). The interRAI home-care assessment is also used across Canada, Europe, some states in the United States, and the Asia-Pacific Rim (Hong Kong, Singapore, Japan, Australia, New Zealand; Morris et al., 2012; Salahudeen & Nishtala, 2019). Other research has demonstrated the feasibility and usefulness of sharing interRAI home-care assessments in improving interprofessional communication and potentially decreasing assessment duplication and workload (Guthrie et al., 2014). The absence of an integrated health record and supportive health information technology negatively influences system capacity to meet the needs of older adults with multiple chronic conditions (Grembowski et al., 2014).

### Study Strengths and Limitations

The diversity of roles of the study participants (i.e., generalist, specialist, advance practice) and their wealth of experience in home care shows representative credibility, in that the results were informed by a variety of sources (Thorne, 2016). Analytic logic is evident in the descriptions of data analysis and findings inclusive of participants’ quotes (Thorne, 2016). Interpretive authority was ensured by the first author’s reflexive journaling, and all authors’ participation in coding, analysis, and writing. Finally, the rationale for undertaking the study—a clinical dilemma without an evidence base to inform a resolution—ensured disciplinary relevance of the study findings.

This study was limited as it captured the experiences of experienced, rather than novice, home-care nurses and was conducted in only three local health integration networks in one province. As well, the perspectives of home-care coordinators were not captured but participants did describe their experiences with those professionals. Also, as the participants’ practice was not observed, the findings are reliant on their accounts of how they practice.

### Implications for Practice

These findings have generated implications for nursing practice. A comprehensive geriatric assessment and holistic treatment plan is required to promote continence and improve T2DM management for home-care clients. A systematic process for educational and consultative opportunities between generalist home-care nurses and those specialized in continence is required in home care. The findings suggest that providing continence education to home-care nurses would be helpful. Many of the components of a continence-promoting intervention are within home-care nurses’ scope of practice (e.g., resolving constipation) and other components (e.g., de-prescribing, addressing financial barriers) could be learned and adopted.

These findings also have implications for research. Further study is required to inform nursing care for older adults with T2DM and UI receiving home-care services. First, this understanding would be enhanced by obtaining the experiences of home-care coordinators and primary-care providers regarding interprofessional collaboration and their perspectives on how to improve communication and coordination of care across settings. Second, this improved understanding could lead to design and testing of strategies to enhance home-care teams’ interprofessional collaboration in the care of clients with T2DM and UI or other complex conditions. Third, the findings from this study will be considered along with the results of the larger study’s quantitative strand and qualitative strand on client experiences to identify the key components of a complex intervention. This newly designed intervention should also be tested in an implementation evaluation.

## Conclusion

These findings enhance understanding of how home-care nurses care for older adults with T2DM and UI. Nurse participants reported using a comprehensive, person- and family-centred approach to the care of older adults that included attending to the complex interplay of multiple chronic conditions with T2DM and UI and the social determinants of health. Nurses reported multiple barriers in providing comprehensive care due to the fragmented and task-focused nature of home care and other health services.

## Supplemental Material

sj-pdf-1-son-10.1177_23779608211020977 - Supplemental material for Home-Care Nurses’ Experiences of Caring for Older Adults With Type 2 Diabetes Mellitus and Urinary Incontinence: An Interpretive Description Study Click here for additional data file. Supplemental material, sj-pdf-1-son-10.1177_23779608211020977 for Home-Care Nurses’ Experiences of Caring for Older Adults With Type 2 Diabetes Mellitus and Urinary Incontinence: An Interpretive Description Study by Melissa Northwood, Jenny Ploeg, Maureen Markle-Reid and Diana Sherifali in SAGE Open Nursing
