# Supplementary material for: Home-Care Nurses’ Experiences of Caring for Older Adults With Type 2 Diabetes Mellitus and Urinary Incontinence: An Interpretive Description Study
Source: SAGE Open Nurs. 2021 Jun 8;7:23779608211020977. doi: 10.1177/23779608211020977 (PMC8193650; doi:10.1177/23779608211020977)
Supplement: sj-pdf-1-son-10.1177_23779608211020977 - Supplemental material for Home-Care Nurses’ Experiences of Caring for Older Adults With Type 2 Diabetes Mellitus and Urinary Incontinence: An Interpretive Description Study [file sj-pdf-1-son-10.1177_23779608211020977.pdf]

Home-care nurses' experiences of caring for older adults with type 2 diabetes mellitus and urinary incontinence: An interpretive description study

Consolidated criteria for reporting qualitative studies (COREQ): 32-item checklist

No.	Item	Location in Text/Comment	Page
1	Interviewer/facilitator	Methods (Data collection)	7
2	Credentials	Methods (Study design)	6
3	Occupation	Methods (Study design)	6
4	Gender	Methods (Study design)	6
5	Experience and training	Methods (Study design) for first author	6, 9
6	Relationship established	Methods (Setting and sampling)	7
7	Participant knowledge of the interviewer	Not stated in text: participants were aware of interviewer's goal of addressing knowledge-practice gap	-
8	Interviewer characteristics	Methods (Study design)	6
9	Methodological orientation and theory	Methods (Study design)	6-7
10	Sampling	Methods (Setting and sampling)	7
11	Method of approach	Methods (Setting and sampling)	7
12	Sample size	Findings (Description of the nurse participants)	9
13	Non-participation	N/A	-
14	Setting of data collection	Methods (Data collection)	7-8
15	Presence of non-participants	N/A	-
16	Description of sample	Findings and Table 2	9
17	Interview guide	Methods (Data collection) and previously published	7
18	Repeat interviews	N/A	-
19	Audio/visual recording	Methods (Data collection)	8
20	Field notes	Methods (Data collection)	8
21	Duration	Methods (Data collection)	7
22	Data saturation	Methods (Data collection)	8
23	Transcripts returned	N/A	-
24	Number of data coders	Methods (Data analysis)	8
25	Description of the coding tree	Methods (Data analysis)	8
26	Derivation of themes	Methods (Data analysis)	8
27	Software	Methods (Data analysis)	8
28	Participant checking	N/A	-
29	Quotations presented	Findings	10-21
30	Data and findings consistent	Findings	10-21
31	Clarity of major themes	Findings and Figure 1	10-21
32	Clarity of minor themes	Findings and Figure 1	

Note. Criteria taken from Tong, Sainsbury, & Craig (2007). N/A = not applicable in this study.
